# The Effect of Thermal Processes on the Organoleptic and Nutraceutical Quality of Tomato Fruit (*Solanum lycopersicum* L.)

**DOI:** 10.3390/foods13223678

**Published:** 2024-11-19

**Authors:** Federica Narra, Federico Ivan Brigante, Eugenia Piragine, Pavel Solovyev, Giada Benedetti, Fabrizio Araniti, Luana Bontempo, Costanza Ceccanti, Alma Martelli, Lucia Guidi

**Affiliations:** 1 Department of Agriculture, Food and Environment, University of Pisa, Via del Borghetto, 80, 56124 Pisa, Italy; federica.narra@phd.unipi.it (F.N.); lucia.guidi@unipi.it (L.G.); 2 Traceability Unit, Research and Innovation Centre, Fondazione Edmund Mach (FEM), Via E. Mach 1, 38098 San Michele all’Adige, Italy; federico.brigante@fmach.it (F.I.B.); pavel.solovyev@fmach.it (P.S.); luana.bontempo@fmach.it (L.B.); 3 Department of Pharmacy, University of Pisa, Via Bonanno, 6, 56126 Pisa, Italy; eugenia.piragine@unipi.it (E.P.); giada.benedetti@phd.unipi.it (G.B.); alma.martelli@unipi.it (A.M.); 4 Interdepartmental Research Center Nutrafood “Nutraceuticals and Food for Health”, University of Pisa, Via del Borghetto, 80, 56124 Pisa, Italy; 5 Dipartimento di Scienze Agrarie e Ambientali—Produzione, Territorio, Agroenergia, Università degli Studi di Milano, 20133 Milan, Italy; fabrizio.araniti@unimi.it

**Keywords:** bioactive compounds, antioxidant activity, intracellular ROS production, lycopene isomers, endothelial cells

## Abstract

The present study investigated the changes in the organoleptic characteristics, nutraceuticals, and antioxidant activity of tomato fruits subjected to different thermal processes: tomato sauce (80 °C for 30 min), blanching treatment (100 °C for 10 s), and the superheated steam method (SHS; 100 °C for 7 min) compared with fresh tomato fruit. Even though SHS negatively modified the color of the product (L* −7% than fresh tomatoes), it was the only technology able to increase the antioxidant activity compared with fresh tomatoes (e.g., +40.3% in ABTS assay), whilst lycopene and ascorbic acid contents reported similar values to fresh tomatoes. Regarding lycopene, only 5Z-lycopene (with a higher bioavailability than (all-*E*)-isomers) was found in all samples, and SHS maintained the same level observed in fresh tomato fruit. Furthermore, SHS technology preserved the antioxidant effects of fresh tomato extract even in human endothelial cells. This result confirmed those obtained in previous “cell-free” assays and demonstrated that SHS treatment significantly maintains the biological properties of tomato fruit in preventing oxidative stress. However, heat-treated tomato extracts did not show the same effects as fresh tomato extract against noradrenaline-induced vasoconstriction in isolated rat aortic rings. This study demonstrates that the use of SHS technology can be considered an innovative and sustainable thermal process (in terms of maintaining the nutraceutical quality) for tomato fruits, thus paving the way for future investigations on the effects of fresh and heat-treated tomatoes after intestinal absorption in vitro and in vivo.

## 1. Introduction

The tomato (*Solanum lycopersicum* L.) is one of the most common vegetables in the Mediterranean area. It is rich in bioactive molecules, including carotenoids (especially lycopene), ascorbic acid, flavonoids (especially naringenin), and some vitamins and minerals [1,2,3]. The complexity of the nutraceutical profile and organoleptic quality of the tomato fruit mainly depends on the growth conditions and ripening stage of the fruit [3].

Tomatoes can be consumed fresh or subjected to different thermal conditions (as reviewed by Wu et al.) [4]. Preserving the nutraceutical profile of tomatoes when subjected to thermal processes is a real and useful challenge to maintain its health effects. Indeed, regular consumption of tomatoes has been associated with a reduction in the risk of cardiovascular diseases (CVDs) [1], which are the leading cause of mortality worldwide [5]. A high intake of fresh tomatoes or tomato sauce (more than 100 g day^−1^; ~5 mg day^−1^ of lycopene) reduced the risk of hypertension by one-third, while a moderate consumption of fresh and processed tomatoes (44–82 g day^−1^; ~2 mg day^−1^ of lycopene) lowered blood pressure levels in patients with hypertension [6]. In addition, the daily intake of tomato juice (200 g day^−1^ for 4 weeks; ~16 ng g^−1^ of lycopene after pasteurization at 80 °C for 60 min) [7], unsalted tomato juice (700 mL day^−1^ for one year; ~0.11 mg mL^−1^ of lycopene) [8], and tomato paste puree (80 g day^−1^ for one week; lycopene content unknown) [9] reduced blood pressure levels in hypertensive patients [6,7,8] and in healthy volunteers [9]. These effects are attributable to the bioactive compounds present in tomatoes, whose retention and biological activity may be affected by thermal processes. Some of the earliest authors who analyzed the effect of different heat treatments on the bioactivity of tomatoes have found that a boiling process (for 15 min) induced a reduction of ascorbic acid, total phenolic and lycopene content, and antioxidant activity when compared with fresh ones, whilst tomatoes baked for 18 min showed a retention of phenols and lycopene content [10]. Dolinsky et al. [11] reported no differences between fresh, boiled, microwaved, pressured, and steamed tomatoes in terms of the content of soluble phenolic compounds, whilst in terms of hydrolysable polyphenol content, microwaved tomatoes showed the lowest value, whereas the steamed tomatoes showed the highest one. These results were similar also for the antioxidant capacity, concluding that steaming was the most recommended method of tomato preparation and microwaving was the least recommended [11].

In addition, other authors focused on the importance of the Z-isomers of lycopene, the most important bioactive molecule in tomatoes, because of their higher bioavailability than (all-*E*)-isomers [12,13]. Honda et al. [13] observed an increase in Z-isomers percentage in tomato products heated at 120 °C for 60 min, with a higher increase in this percentage in oil-containing products such as tomato oleoresin, ketchup, or pizza sauce. In recent years, the Z-isomers of lycopene in tomatoes have been analyzed by nuclear magnetic resonance (NMR) [14]. Honda et al. [14] observed that an increase in temperature (from 50 to 70 °C) induced an increase in Z isomerization, especially for 9Z- and 13Z-isomers, confirming the results of the previous works by the same authors [12,13]. Moreover, they showed that adding plant foods such as onion, broccoli, mustard, makonbu, or shiitake mushrooms to tomato pulp promoted the Z-isomerization of lycopene.

The search for new heating technologies able to preserve the bioactivity of tomatoes is a current challenge. In this field, the use of superheated steam (SHS) technology is a recent introduction to obtain roasted vegetables [15,16,17,18]. This technology involves heating steam to temperatures above its boiling point at a given pressure, resulting in a state where this heating steam contains more energy than saturated steam at the same pressure [19]. This higher energy allows SHS to transfer more heat to the plant material. SHS can create a low-oxygen environment [16,20], thus preventing lipid peroxidation and the formation of harmful compounds [21] and minimizing nutrient oxidation and their consequent loss [21]. For example, Ceccanti et al. [16] observed an increase in antioxidant activity, ascorbic acid, and total carotenoid content in artichokes roasted at 100 °C for 6 min using SHS, and Shaharuddin et al. [17] noted an increase in total phenolic content and antioxidant activity in Kejirak fruit supersteamed at 170 °C for 15 min.

Given the conflicting results on the retention of bioactive compounds in tomato products subjected to different thermal processes, as reviewed by Wu et al. [4], and the complete lack of information on the use of SHS technology on tomatoes, the first aim of this study was to standardize the knowledge about the effect of different heat treatments on the retention of the organoleptic and nutraceutical qualities of tomato fruit. The evaluation of the level of isomerization of (all-*E*)-isomers in Z-isomers provided further standard insights into the bioavailability of lycopene in heat-treated tomato fruit. Finally, the impact of thermal processes on the biological properties of tomato fruit extracts was evaluated in human endothelial cells to provide a basis for future investigations into the potential effects of heat treatments on the vascular effects of tomatoes.

## 2. Materials and Methods

### 2.1. Materials

In July 2023, tomato fruits (*S. lycopersicum* L. var. *Big Rio*) were purchased from a local farmer located in Campiglia Marittima (Livorno, Italy) and supplied by a specialized nursery (Orto Mio, Forlì, FC, Italy). All chemical reagents were purchased from Merck KGaA (Darmstadt, Germany).

### 2.2. Plant Material

The tomatoes were previously selected by the same operator based on their uniform red color (the average parameter a* (red-green) was 19.71 ± 3.63) to ensure a homogeneous representation of the product. Samples were prepared by dividing all tomato fruit into four pools, each consisting of thirty tomatoes, with one pool assigned to each treatment and one representing the control (fresh plant material; Cnt). Each pool was subdivided into three biological replicates (n = 10 fruits per replicate).

### 2.3. Heat Treatments

Thermal processes were carried out with professional kitchen tools, and the parameters were decided basing on pre-experiments of the heat treatments, which are summarized in Table 1.

To prepare the tomato sauce (TS), the tomatoes were cut into several small pieces and placed on a stove; the mixture was heated for ~30 min using a temperature probe to monitor the temperature in the middle of the TS sample. The TS treatment was stopped when the samples reached a great balance between sauce and water (the moisture average was 91.5%) The blanching treatment (BL) was performed by immersing tomatoes (one by one) on a stove with boiling water (1 L) at 100 °C for 10 s, based on the edibility of fruit tomatoes. At the end of the treatment, the tomato samples were placed on ice for 1 min, and the skin was removed. SHS was carried out employing a commercially available professional oven provided by ATIHC Srl (Legnago, Verona, Italy). This professional electric steam oven had a maximum capacity of six trays, a chamber dimension of 955 mm x 878 mm x 818 mm, and a maximum power of 12 kW. This oven develops SHS from tap water and allows the use of a temperature range of 100–500 °C. The firing process was automated to keep a constant temperature of 100 °C in the firing chamber, and the treatment duration was 7 min, based on the edibility of the fruit tomatoes.

After the heat treatments, each sample replicate (from treated and control pool) was subdivided into two portions; one was used to perform organoleptic analysis, and the other was ground using an A11 basic analytical mill (IKA-Werke GmbH & Co. KG, Staufen, Germany). The samples were immediately frozen with liquid nitrogen and stored at −80 °C until biochemical analysis.

### 2.4. Dry Matter, Color Measurements, Soluble Solid Content (SSC), and Titratable Acidity (TA)

Fresh and heat-treated tomatoes were dried (n = 3) in an electric thermostatic laboratory oven (Memmert GmbH + Co. KG Universal Oven UN30, Schwabach, Germany) at 105 °C for 48 h until the samples reached a constant weight, and the percentage of dry matter was calculated using the following equation:

Dry matter (%) = (DW/FW) × 100

where DW was the dry weight of the tomato, and FW was the fresh weight of the tomato.

Color measurements (n = 12) were performed on fresh and heat-treated tomato samples. The color changes were measured in the CIELab color space using a portable Konica Minolta spectrophotometer model CM 1006d (Konica Minolta Holdings, Inc., Osaka, Japan). Before the measurements, the instrument’s 8 mm diameter head was calibrated to the appropriate white etalon. Brightness (L*), redness (a*), and yellowness (b*) were recorded.

The SSC in tomato samples (n = 9) was analyzed using an ATAGOTM PAL-1 (Thermo Fisher Scientific Inc., Milan, Italy) digital refractometer. A drop of juice obtained by squeezing the tomato fruit was placed on the reader of the digital refractometer, which provided data in °Brix describing the mass fraction of sucrose in solution (1 °Brix equals 1 g sucrose per 100 g of aqueous solution). SSC was expressed as %.

TA (n = 3), representing the total organic acids in solution in tomato samples, was determined using an acid/base titrator. To 1 g of tomato fruit homogenate, 30 mL of distilled water was added and titrated to neutral with a 0.1 N sodium hydroxide solution. TA was expressed as the percentage of the acid most represented in the fruit, which in the case of tomato is citric acid (% citric acid).

### 2.5. Determination of Lycopene Content

The lycopene content (n = 3) was measured as described by Adejo [22] with some adjustments. An amount of 0.1 g FW of ground tomato samples was extracted in 1 mL of distilled H_2_O and incubated in a water bath for 1 h at 30 °C. Afterwards, 8 mL of a hexane/ethanol/acetone mixture (2:1:1; *v*:*v*:*v*) was added to each sample, and the mixture was vortexed and incubated in the dark for 10 min. Then, 1 mL of distilled H_2_O was added; each sample was vortexed and incubated for 10 min until the mixture was separated into two phases. The absorbance of 1 mL of the upper phase of samples was measured spectrophotometrically (Ultrospec 2100 Pro, GE Healthcare Ltd., Little Chalfont, Buckinghamshire, United Kingdom) at 503 nm compared with a blank solution containing only a hexane/ethanol/acetone mixture (2:1:1; *v*:*v*:*v*) and distilled H_2_O. The lycopene content in the extracts was calculated using the following equation described by Gisbert-Mullor et al. [23]:

Lycopene (mg kg^−1^ DW) = {[(Abs_503_/172,000 × 1 cm) × (10 mL/1000) × 0.55] × 537 g mol^−1^} × 1000 × DW

where Abs_503_/172,000 M^−1^ cm^−1^ is the Lambert–Beer equation’s resolution, and 172,000 L (mol × cm)^−1^ is the molar extinction of lycopene (in hexane); 0.55 is the correction factor resulting from the ratio between the 8 mL of the hexane/ethanol/acetone mixture and the upper phase containing only hexane; 10 mL/1000 is the volume of the final mixture converted in L; 537 g mol^−1^ is the molecular weight of lycopene and DW is the dry weight of the samples expressed in kg. The lycopene content of the extracts was calculated as mg g^−1^ DW.

### 2.6. Determination of Total Phenolic Content (TPC)

The TPC (n = 3) was measured according to Ceccanti et al. [24]. Briefly, 0.1 g of ground tomato material was homogenized in 1 mL of 80% (*v/v*) methanolic solution and centrifuged with a centrifuge (MPW-260R, MWP Med. Instruments, Warsaw, Poland) at 10,000× *g* for 15 min at 4 °C. A total of 10 μL of the supernatant was added to a solution containing 115 μL of distilled water, 125 μL of Folin-Ciocalteu reagent, and 1.25 mL of a 7% (*w/v*) Na_2_CO_3_ aqueous solution. The oxidative breakdown of phenolic compounds and the reduction of metals in the solution of phosphomolybdate/phosphotungstate of Folin–Ciocalteu reagent, as well as the resulting color blue, were spectrophotometrically detected at 760 nm. The measurements were compared with a gallic acid standard curve (y = 0.002x + 0.0008; R^2^ = 0.9934), and TPC was expressed as mg gallic acid equivalents (GAEs) per g DW.

### 2.7. Determination of Total Ascorbic Acid Content (AscA)

The AscA (n = 3) was calculated following the methodology described by Garcìa-Martinez et al. [25]. A total of 0.1 g of ground tomato material was homogenized in 1 mL of 6% (*w*/*v*) trichloroacetic acid (TCA) and centrifuged at 14,000× *g* for 10 min at 4 °C. The supernatant was immediately utilized for converting the dehydroascorbate to ascorbate by pre-incubating 50 µL of the extract with 50 µL of 10 mM dithiothreitol (DTT) and 100 µL of Na-P buffer (0.2 M; pH 7.4). The mixture was incubated for 10 min in the dark at room temperature. The excess of DTT was removed by adding 50 µL of 0.5% (*w*/*v*) N-ethylmaleimide (NEM) solution, and the mixture was vortexed vigorously for 1 min. Subsequently, 250 µL of 10% (*w*/*v*) TCA solution, 200 µL of 42% (*w*/*v*) H_3_PO_4_ solution, 200 µL of 4% (*w*/*v*) 2,2′-dipyridil solution, and 100 µL of 3% (*w*/*v*) FeCl_3_ solution were added, and the samples were incubated for 40 min at 42 °C. The absorption of the final solution was measured spectrophotometrically at 525 nm and AscA was expressed as μg g^−1^ DW, using an ascorbic acid standard calibration curve (y = 0.0147x − 0.0042; R^2^ = 0.9954).

### 2.8. Antioxidant Activity Determination

To determine the antioxidant activity, the extracts prepared for the quantification of TPC were used for two different assays: the 2,2-diphenyl-1-picrylhydrazyl (DPPH) free radical scavenging assay described by Ceccanti et al. [26], and the 2,2′-azino-bis (3-ethylbenzothiazoline-6-sulfonic acid) (ABTS) radical cation-based assay, performed according to the method described by El Horri et al. [27].

To perform the DPPH assay (n = 3), 10 μL of tomato extract was added to 990 μL of 80% (*v/v*) methanolic solution containing 3.12 × 10^−1^ M DPPH (*w/v*). In this method, 30 min of incubation in the dark at room temperature was required to observe the interaction between the antioxidant compounds present in the samples and the DPPH radical. The antioxidant compounds reduced the violet DPPH radical, resulting in color loss (from violet to pink). The color loss was evaluated spectrophotometrically at 515 nm compared with a blank solution (without sample extracts). Each measurement was compared to a Trolox standard curve (y = 0.0045x − 0.0002; R^2^ = 0.9915), and antioxidant activity was expressed as mg Trolox equivalents (TE) per g of DW.

In the ABTS assay (n = 3), 50 µL of tomato extract was mixed with 950 µL of ABTS solution (at least 16 h before performing the assay), prepared by adding 7 mM (*w/v*) ABTS and 2.5 mM (*w/v*) K_2_S_2_O_8_ in 5 mL of Na-P buffer 5 mM (*w/v*). Subsequently, the kinetics of the reaction were spectrophotometrically monitored for 90 s at 734 nm. Results were expressed as mg TE g^−1^ DW, following the standard curve y = 0.0012x + 0.0246; R^2^ = 0.9926.

### 2.9. Sample Preparation for ^1^H NMR Analysis

Ground tomato samples were lyophilized for 48 h before NMR analysis. Briefly, 150 mg of each lyophilized sample (fresh and three thermal treatments; n = 3) were mixed with 1.3 mL of deuterated chloroform (CDCl3) with tetramethylsilane (TMS) for internal reference (Chloroform-d, 99.8% + Ag + 0.03% TMS, Deutero GmbH, Kastellaun, Germany) in an Eppendorf tube. The samples were ultrasonicated for 30 min (ultrasonic peak power, 320 W, rated ultrasonic power, 80 W). Then, samples were centrifuged for 15 min at 18,000× *g* at room temperature (5804R, Eppendorf, Hamburg, Germany). Each extract was filtered with a single-use all-plastic syringe (fisherbrand 2 mL, unsterile, Fisher Scientific, Schwerte, Germany) and filtering unit (Low protein binding Durapore^®^, PDVF, 0.22 μm, Millex^®^, 13 mm, Merck Millipore, Wicklow, Ireland). Finally, 600 µL of the extract was transferred into an NMR tube (Norell^®^ Standard Series TM, 5 mm, Sigma-Aldrich, St. Louis, MO, USA).

### 2.10. NMR Spectroscopy and Identification of Lycopene Isomers

All the ^1^H NMR measurements were recorded on a Bruker Avance Neo 600 spectrometer (base frequency 600 MHz ^1^H), with a broadband Z-gradient probe (5 mm sample tubes) and SampleCase autosampler of 24 positions (Bruker BioSpin GmbH, Rheinstetten, Germany). The spectra were acquired and processed using Topspin 4.1.1 software in automation mode with Icon NMR 5.2.1. The size of each spectrum (sweep width, SW) was 15.22 ppm, the relaxation delay (D1) of 10 s, the 90-degree pulse width was 8.65 µs, the number of scans (NS) was 128, and the number of dummy scans (DS) was 2. The acquisition time was 2.7525 s, the acquired size (TD) was 65,536 points, the receiver gain (RG) for all spectra was fixed at 64, and baseopt digitization mode was used. Automatic adjustment of the probe (ATMA routine) and automatic shimming (TOPSHIM) were performed before acquisition. Identification of the E and Z isomers of lycopene was performed manually by comparison with bibliographic sources.

### 2.11. Quantification of 5Z-Lycopene

Quantitative analysis was performed using Assure-NMR software (2020.09.23) with an external standard technique (ERETIC or Electronic Reference To access In vivo Concentrations) [28], with an ethylbenzene solution in CDCl_3_ used as the external standard (concentration: 8.162 mM). All the spectra (n = 3) were pre-processed before quantification. Baseline correction and 0th and 1st order phase correction were applied with built-in commands in NMR processing software (TopSpin 4.3.0 with IconNMR 6.1.0, Bruker, TopSpin). The concentration of lycopene isomers in tomato samples was calculated in mg g^−1^ DW. Specificity was assessed by checking that the baseline around the signal of interest (the triplet at 2.24 ppm) was flat and straight. The signal to be quantified was the only signal in the region from 2.18–2.26 ppm. It only presented one deviation in one of the replicates, which was excluded from the statistical analyses. The spectra of the external standard and the quantified signal in the extracts are presented in Figures S1 and S2 (see Supplementary Material File), respectively. It has been reported that a minimum signal-to-noise ratio (S/N) of 15:1 must be achieved to quantify within 1% of uncertainty [29]. Samples with S/N below this threshold were excluded from the analysis. The accuracy was obtained by calculating the percentual relative standard deviation (% RSD) of the integrated signal across all the included replicates (n = 33). The S/N ratio of the replicates, the analysis date, and the class and total % RSD of the quantified signal are reported in Table S1 (see Supplementary Material File).

### 2.12. Cell Cultures

Human aortic endothelial cells (HAECs; Lonza Bioscience, Basel, Switzerland) were cultured in Endothelial Cell Growth Basal Medium-2 (EBM-2; Lonza Bioscience, Basel, Switzerland) enriched with fetal bovine serum (FBS), hydrocortisone, human fibroblast growth factor (hFGF), vascular endothelial growth factor (VEGF), a recombinant analog of insulin-like growth factor (R3-IGF-1), ascorbic acid, human epidermal growth factor (hEGF), gentamicin (30 mg mL^−1^), amphotericin (15 µg mL^−1^), and heparin (all from Lonza Bioscience, Basel, Switzerland). Streptomycin (100 mg mL^−1^) and penicillin (100 U mL^−1^) (Merck KGaA, Darmstadt, Germany) were added to avoid bacterial contamination. Cells were grown in a CO_2_ (5%; *v/v*) incubator at 37 °C in T-75 flasks and used between passages 5–7.

### 2.13. Animal Procedures

The experiments were carried out in accordance with the Code of Ethics of the World Medical Association (Declaration of Helsinki, EU, Directive 2010/63/EU for animal experiments) and the guidelines of the European Community Council Directive 86-609. The experiments were authorized by the Italian Ministry of Health (authorization number DB173.N.IXS). Animals were housed in humidity- and temperature-controlled rooms (50% and 22 °C, respectively) with 12 h light/dark cycles, water, and food ad libitum (diet composition: 18.6% crude protein, 6.2% fat, 44.2% carbohydrate, 3.5% crude fiber, 14.7% neutral detergent fiber; Envigo, Indianapolis, IN, USA). Animal studies were conducted following ARRIVE guidelines and the Basel Declaration, including the 3Rs concept [30,31]. All procedures were performed to minimize the number of animals used and their pain.

### 2.14. Preparation of Tomato Extracts for Experiments on Endothelial Cells and Isolated Rat Aorta

One mL of dimethyl sulfoxide (DMSO; Merck KGaA, Darmstadt, Germany) was added to 50 mg of lyophilized tomato samples. The mixture was vortexed, incubated at room temperature in the dark on a rotary shaker for 15 min, and then centrifuged for 10 min at 10,000× *g* at room temperature. The pellets were discarded, and the supernatant was used for experiments on cultured endothelial cells and isolated rat aortic rings (see below). This procedure allowed us to avoid using solvents other than DMSO, which are toxic to biological samples, without losing the content of total phenolics and lycopene. The mixtures were freshly prepared on the day of the experiment and diluted in culture medium (for in vitro assays) or Tyrode solution (for ex vivo assays).

### 2.15. Measurement of Intracellular ROS Production in Endothelial Cells

HAECs were seeded in a 96-well black plate (30,000 cells/well) and pre-coated with an aqueous solution of gelatin (1% *w/v*). The next day, the culture medium was removed, and fresh medium containing vehicle (DMSO 0.25%; *v/v*) or tomato extracts (0.125 µg mL^−1^) was pre-incubated for 1 h. Then, the cells were treated with hydrogen peroxide (H_2_O_2_, 200 µM) for 2 h. At the end of the treatment, the medium was replaced with a freshly prepared dihydroethidium (DHE; Merck KGaA, Darmstadt, Germany) solution (10 µM). The plate was incubated in a CO_2_ incubator in the dark at 37 °C for 30 min to allow the probe to react with intracellular superoxide and generate a fluorescent product. Fluorescence was measured with the EnSpire microplate reader (PerkinElmer, Shelton, CT, USA) (λex = 500 nm; λem = 580 nm) [32]. Experiments were performed in triplicate at least twice (n = 6).

### 2.16. Preventive Effects Against Noradrenaline (NA)-Induced Vasoconstriction in Isolated Rat Aortic Rings

Three-month-old male Wistar rats, housed in cages with free access to food and water, were anesthetized with an intraperitoneal injection of sodium thiopental (MSD Animal Health, Milan, Italy; 100 mg kg^−1^) and the descending thoracic aorta segment was excised, cleaned, and cut into 5 mm aortic rings. After removing the endothelial layer by rubbing the intima surface of the vessels with a hypodermic needle, the aortic rings were suspended, with a pre-load of 2 g, in chamber baths (ADInstruments, Dunedin, New Zealand). Each bath contained 20 mL of Tyrode solution (composition: NaCl 136.8 mM; KCl 2.95 mM; CaCl_2_ × 2H_2_O 1.80 mM; MgSO_4_ × 7H_2_O 1.05 mM; NaH_2_PO_4_ × H_2_O 0.41 mM; NaHCO_3_ 11.9 mM; glucose 5.5 mM). The temperature of the baths was set at 37 °C, and the solution was continually gassed with Clioxicarb (95% O_2_ and 5% CO_2_; *v/v*) to replicate physiological conditions. An isometric transducer combined with an amplifier and LabChart Pro software 8 (ADInstruments, Dunedin, New Zealand) recorded the voltage changes.

After 30 min of stabilization, endothelium removal was confirmed by inducing vasorelaxation with acetylcholine (Ach, 10 μM; Merck KGaA, Darmstadt, Germany) on aortic rings pre-contracted with KCl 25 mM. A relaxation of < 10% was considered acceptable. Aortic rings were washed and incubated with vehicle (DMSO 2.5%) or tomato extracts (1.25 mg mL^−1^) for 20 min. Then, increasing concentrations of noradrenaline (NA, 10^−9^ M–10^−6^ M) were added in each bath to induce vasoconstriction. Once the NA concentration-response curve was obtained, the aortic rings were washed, and after a stabilization period of 20 min, KCl 60 mM was added to achieve maximum contraction (100%). The vasoconstrictor effect of NA was expressed as a percentage (%) of the maximum contractile tone induced by KCl 60 mM [33]. The results, expressed as the maximum vasoconstriction obtained after the administration of the maximum concentration of NA (Emax), are expressed as mean ± standard error of the mean (SEM) of six experiments (n = 6) for each different treatment.

### 2.17. Statistical Analysis

All the “cell-free” experiments were performed using a completely randomized design with different replicates depending on the bioassay, as reported in the paragraph of each specific assay. The data are represented as the mean ± standard deviation (SD). Data from the organoleptic and biochemical analyses were examined using one-way analysis of variance (ANOVA), considering the different heat treatments as the variability factor. Means were separated using Fisher’s least significant difference (LSD) post-hoc test (*p* ≤ 0.05). The hypothesis of homogeneity of variances was investigated using Bartlett’s test, whilst the normality of data was evaluated using the Shapiro–Wilk test. Statistical analyses were performed using GraphPad software 9.0 (GraphPad, La Jolla, CA, USA).

In addition, all the analyses regarding NMR spectroscopy were performed using R Statistical Software v4.3.1; R Core Team [34] with the tidyverse, ggpubr, and rstatix packages. The mean (in mg g^−1^ DW) and SD were calculated for fresh and heat-treated samples. Shapiro–Wilk and Levene’s tests were applied to assess the normality and homogeneity of variances between all the samples. Then, a one-way ANOVA was performed to find statistically significant differences between fresh and heat-treated samples. Pairwise comparison tests (Tukey’s post-hoc test), in the case of statistically significant results, were performed to discuss the impact of thermal processes further.

Data from the experiments on cultured endothelial cells and isolated rat aortic rings were expressed as mean ± SEM and analyzed using one-way ANOVA followed by Bonferroni’s post-hoc test. Statistical analysis was performed with GraphPad Prism software 9.0 (GraphPad, La Jolla, CA, USA) and statistical significance was set at *p* ≤ 0.05.

## 3. Results

### 3.1. Tomato Organoleptic and Nutraceutical Quality

**Figure 1:**
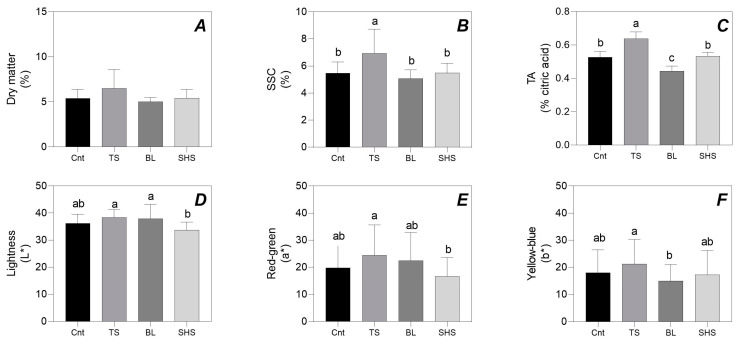
Dry matter (**A**), soluble solid content (SSC; (**B**)), titratable acidity (TA; (**C**)), lightness (**D**), redness (**E**), and yellowness (**F**) of fresh tomato fruit (Cnt), or subjected to sauce preparation (TS), blanching (BL), and superheated steaming (SHS). Bars with the same letter are not significantly different for *p* = 0.05 following one-way ANOVA using the different cooking treatments as the variability factor. Bars with different lowercase letters are significantly different after Fisher’s LSD post-hoc test (*p* ≤ 0.05).

Figure 1 summarizes the values obtained from the organoleptic evaluations of fresh (Cnt) and heat-treated tomato fruit.

Dry matter percentage showed no differences between heat-treated tomatoes and Cnt (Figure 1A). The tomato sauce had the highest SSC (+21% than Cnt), whilst all other thermal processes induced a similar SSC to Cnt tomatoes (Figure 1B). TS also showed the highest TA (+16%), whilst BL induced the lowest TA (−17%; Figure 1C). Color measurements revealed that SHS tomatoes had lower lightness than TS and BL but reported similar values compared to Cnt tomatoes (Figure 1D). SHS also induced a lower redness when compared with TS but with similar results when compared to Cnt tomatoes and the other thermal processes (Figure 1E). In contrast, BL induced a lower yellowness when compared to TS but with similar results when compared to Cnt tomatoes and the other thermal processes (Figure 1F). TS revealed the highest values of both redness and yellowness parameters (Figure 1E,F).

**Figure 2:**
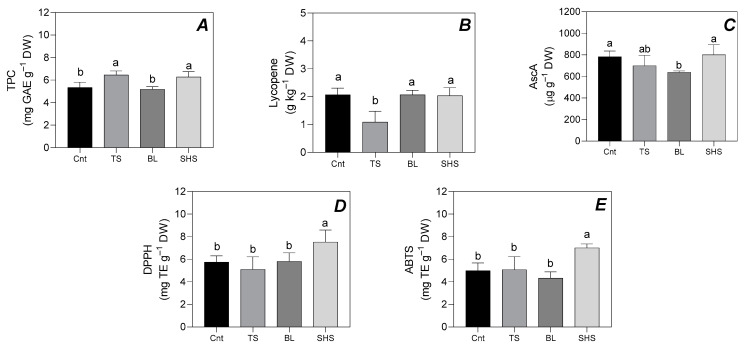
Total phenolic content (TPC; (**A**)), lycopene content (**B**), total ascorbic acid content (AscA; (**C**)), and antioxidant activity assayed by the DPPH (**D**) and ABTS (**E**) methods of fresh tomato fruit (Cnt), or subjected to sauce preparation (TS), blanching (BL), and superheated steaming (SHS). Data were analyzed through one-way ANOVA using the different cooking treatments as the variability factor. Bars with different lowercase letters are significantly different after Fisher’s LSD post-hoc test (*p* ≤ 0.05).

Figure 2 shows the TPC, lycopene, AscA, and antioxidant activity of fresh and heat-treated tomato fruit.

A significant increase in TPC was observed in TS and SHS tomato fruit when compared to Cnt and BL tomatoes (Figure 2A). Conversely, a significant decrease (−47%) in lycopene content was observed in TS samples when compared to Cnt, whilst all the other thermal processes revealed a similar lycopene content to Cnt tomatoes (Figure 2B). Furthermore, Figure 2C shows that AscA significantly decreased by −18.5% in BL tomato samples when compared to Cnt and by −20.3% when compared to SHS. The antioxidant activity showed a significant increase only in SHS tomato samples. This trend was observed in both the DPPH and ABTS assays (+31% and +40.3%, respectively; Figure 2D,E).

### 3.2. Dentification of E and Z Isomers of Lycopene in Tomato Fruit

**Figure 3:**
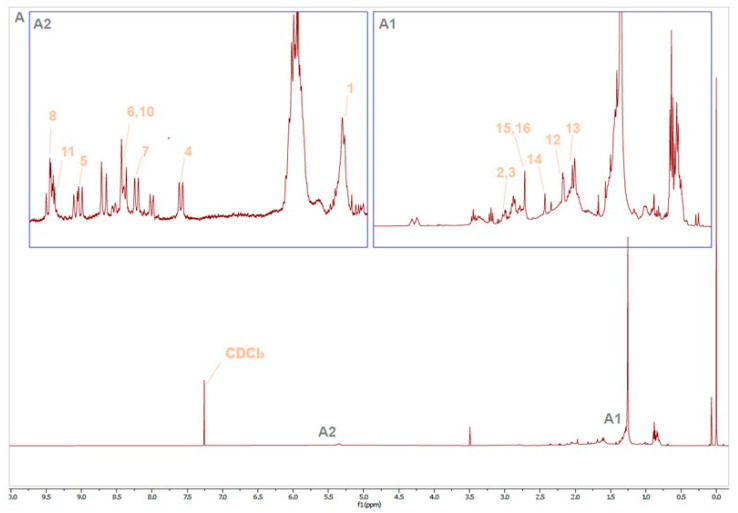
^1^H NMR assignment of (all-*E*)-lycopene isomers from fresh tomato samples with the expansion of the region from 0 to 3 ppm (panel **A1**) and the expansion of the region from 5.5 to 7.4 ppm (panel **A2**).

Firstly, the identification of (all-*E*)-lycopene was carried out in Cnt freeze-dried tomatoes. The assignment of (all-*E*)-lycopene is presented in Table 2 and Figure 3.

The spectrum was clearly divided into two regions. The resonances of the methyl groups were observed in the aliphatic region from 0.6 to 2.0 ppm. Particularly, methyl groups in carbons 16, 17, and 18 could be clearly distinguished by their singlets at 1.615, 1.819, and 1.969 ppm, respectively (Figure 3, panel A1). This was not the case of methyl groups in carbons 19 and 20, which appeared together as a broad singlet, while other authors achieved their clear identification as two singlets in C_6_D_6_ from extraction and purification by the recrystallization of (all-*E*)-lycopene isomers from tomatoes [35]. Moving towards the low-field region of the spectrum, protons from carbons 2 and 4 appeared as a multiplet with a center at 2.11 ppm, and in this case, discrimination based on the chosen solvent was not possible (Figure 3, panel A1).

The other group of signals of interest in the mixture was presented in the low-field region from 5.0 to 6.7 ppm and belonged to vinylic protons (Figure 3, panel A2). The doublet at 5.95 ppm showed the typical coupling constant value for a trans-partial double bond (11.0 Hz) between C6 and C7, and C6′ and C7′ [36]. In the case of protons 7, 7′ and 11, 11′ a doublet of doublets (dd) could be clearly observed. In the case of protons from carbon 7, the coupling constants could be perfectly calculated and matched those from the bibliography. However, in the case of protons from carbon 11, only the top part of the signal was observed, and a close inspection of the zone revealed the presence of bumps along the signal and typical overlapping observed in complex mixtures. This caused the coupling constant to be different from the reported values (Figure 3, panel A2).

**Figure 4:**
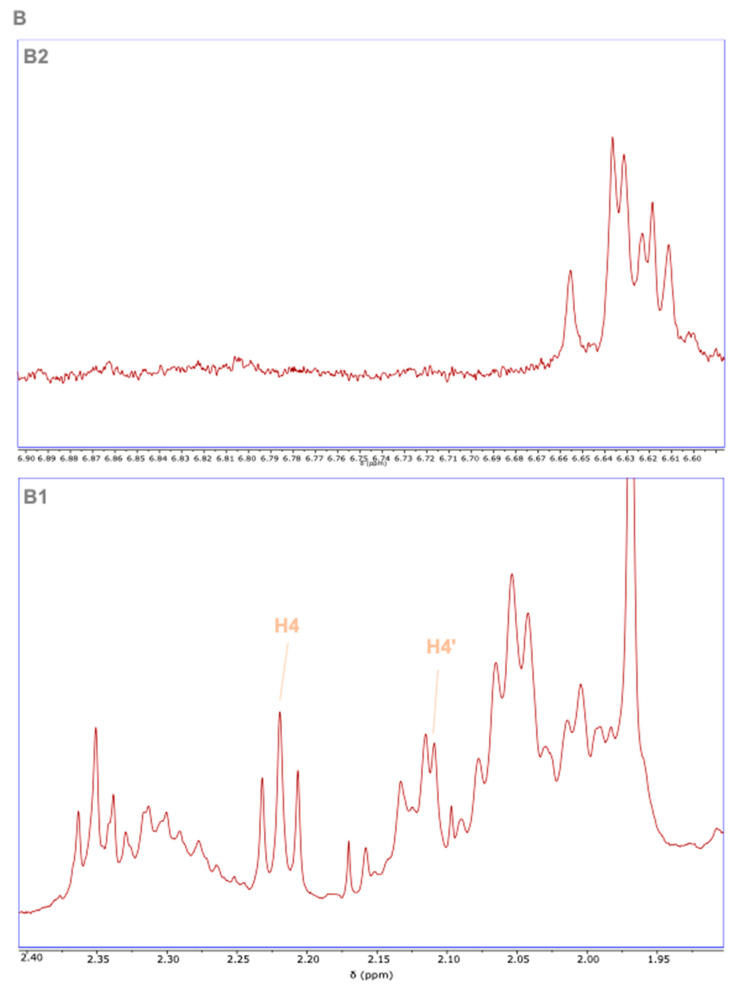
^1^H NMR assignments of Z isomers in fresh tomato samples with the expansion to show the triplet from H-4 at 2.22 ppm (panel **B1**) and the expansion to show the absence of specific signals from isomers 9Z and 13Z (panel **B2**).

The search for the three most present Z isomers (5Z, 9Z, and 13Z) was carried out in Cnt and heat-treated samples (Figure 4).

The 13Z isomer has a characteristic signal (a multiplet) from proton 12 at 6.88 ppm, which was not observed in our extracts (Figure 4, panel B2). Then, isomer 9Z is characterized by the presence of a doublet at 6.70 ppm from proton 8, which was also absent in our extracts (Figure 4, panel B2). Finally, the protons from carbon 4 in (all-*E*)-lycopene isomers that appear as a multiplet at 2.11 ppm come with different shifts after isomerization. 5Z-lycopene could be distinguished by the triplet from protons in carbon 2 at 2.22 ppm with a coupling constant of 7.6 Hz belonging to proton 4, in agreement with the bibliography (Figure 4, panel B1), while proton 4′ remained at the same chemical shift at 2.11 in 5Z-lycopene. It has been demonstrated by means of 2D NMR techniques like T-ROESY and TOCSY that this signal at 2.22 ppm belongs exclusively to this isomer [37]. This signal showed a good intensity with a signal-to-noise ratio higher than 100, it did not overlap with any other resonances in the spectrum, and it was well-resolved, so 5Z-lycopene was quantified in all the extracts to assess the impact of different heat treatments.

### 3.3. Quantification of 5Z-Lycopene in Tomato Fruit

**Figure 5:**
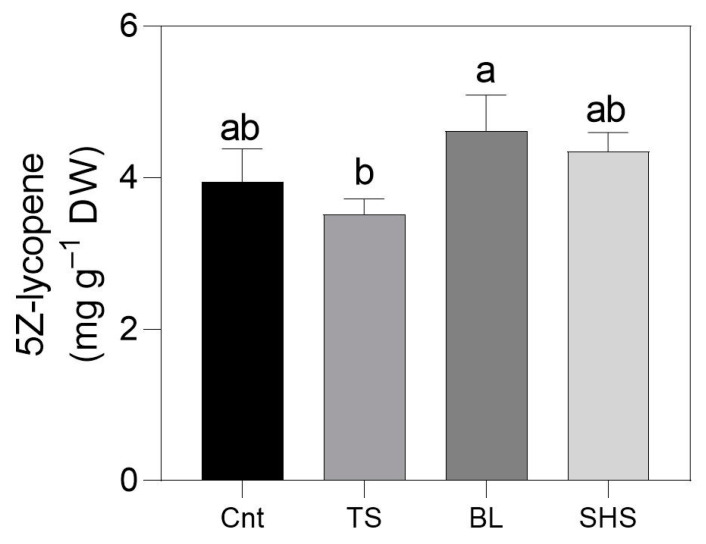
*5Z*-lycopene content of fresh tomato fruit (Cnt) or subjected to sauce preparation (TS), blanching (BL), and superheated steaming (SHS). Bars with the same letter are not significantly different for *p* ≤ 0.05 following one-way ANOVA using the different cooking treatments as the variability factor. The means were separated by Tukey’s post-hoc test.

The results of the concentration of 5Z-lycopene with the statistically significant differences in Cnt tomatoes and fruit subjected to all heat treatments are presented in Figure 5.

The obtained S/N ratio values and % RSD confirmed reproducibility and good precision, and that all the signals included in the analysis were quantified within the threshold of uncertainty. It could be observed that TS had the lowest concentration of 5Z-lycopene with a decrease in content (−23.8%) when compared with BL tomatoes. Conversely, BL induced the highest 5Z-lycopene content but reported similar results to Cnt tomatoes as well as SHS.

### 3.4. Preventive Effects of Tomato Extracts Against Intracellular ROS Production in Endothelial Cells

Based on the results obtained in “cell-free” assays, the potential impact of different thermal processes on the preventive effects of tomato fruit extracts against an oxidative stimulus (H_2_O_2_) was evaluated in cultured endothelial cells (HAECs). In fact, the endothelium is the first layer of the blood vessel to interact with circulating oxidative mediators under oxidative stress conditions and with bioactive compounds after food consumption.

**Figure 6:**
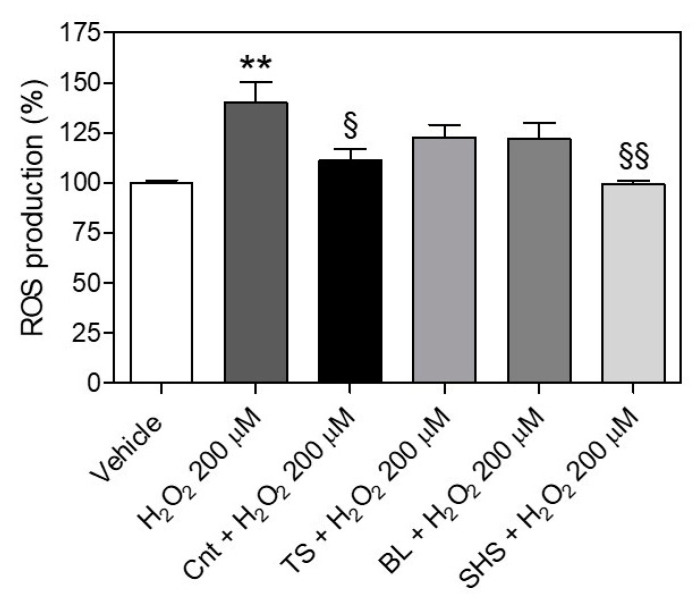
Preventive effects of tomato fruit extract against intracellular ROS production in HAECs. Bars indicate ROS production (%) in HAECs treated with tomato fruit (0.125 µg mL^−1^) or vehicle (DMSO 0.25%) for 1 h, and then exposed to H_2_O_2_ 200 µM for 2 h. Pre-incubation of fresh tomato fruit extract (Cnt) and tomato fruit subjected to superheated steaming (SHS), but not to sauce preparation (TS) or blanching (BL), prevented H_2_O_2_-induced ROS production. The statistical analysis was one-way ANOVA followed by Bonferroni’s post-hoc test. ** means significantly different from vehicle (*p* ≤ 0.01); § means significantly different from H_2_O_2_ 200 µM (§ *p* ≤ 0.05; §§ *p* ≤ 0.01).

Incubation of H_2_O_2_ 200 µM for 2 h significantly increased intracellular ROS levels in HAECs (% ROS vs. vehicle: 140.0 ± 22.9) (Figure 6).

Pre-treatment with fresh tomato extract (Cnt) and tomato subjected to SHS treatment, but not to TS or BL, significantly prevented H_2_O_2_-induced ROS production (% ROS vs. vehicle: 111.0 ± 11.9 for Cnt, 99.2 ± 3.3 for SHS, 122.7 ± 12.7 for TS and 121.7 ± 17.9 for BL).

### 3.5. Preventive Effects Against NA-Induced Vasoconstriction in Isolated Rat Aortic Rings

Given the antioxidant activity promoted by tomato fruit in cultured endothelial cells and the potential anti-hypertensive properties described for tomatoes, the preventive effects of tomato fruit extracts against a vasoconstrictive stimulus (i.e., NA) were evaluated in isolated rat aortic rings. Indeed, the prevention of oxidative stress is a recognized, though not the only, mechanism responsible for the vasoprotective effects of many compounds.

**Figure 7:**
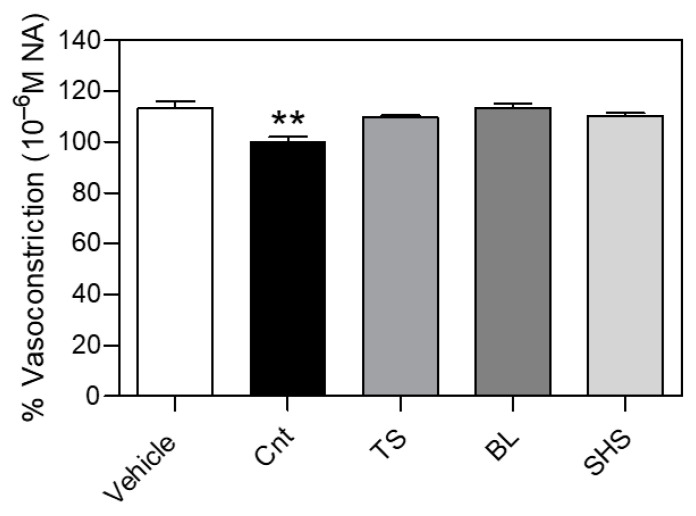
Preventive effects of tomato fruit extract against noradrenaline (NA)-induced vasoconstriction in isolated rat aortic rings. Bars indicate the Emax (maximum vasoconstriction %) of isolated rat aortic rings incubated with tomato fruit (12.5 mg mL^−1^) or vehicle (DMSO 2.5%) for 20 min, and then treated with growing concentrations of NA (10^−9^–10^−6^ M). Pre-incubation of fresh tomato fruit extract (Cnt), but not tomato fruit subjected to sauce preparation (TS), blanching (BL), or superheated steaming (SHS), prevented NA-induced vasoconstriction. The statistical analysis was one-way ANOVA followed by Bonferroni’s post-hoc test. ** means significantly different from vehicle (*p* ≤ 0.01).

In this series of ex vivo experiments, the highest concentration of NA tested (10^−6^ M) led to a marked vasoconstriction of the aortic rings (% vs. KCl 60 mM: 113.1 ± 2.9) (Figure 7).

Only the pre-incubation of fresh tomato fruit extract for 20 min significantly prevented NA-induced vasoconstriction, whereas tomato fruit subjected to different thermal processes (i.e., SHS, TS, and BL) showed no effect (% vs. KCl 60 mM: 100.0 ± 1.9 for Cnt, 110.3 ± 1.1 for SHS, 109.7 ± 0.6 for TS and 113.3 ± 1.7 for BL).

## 4. Discussion

### 4.1. The Impact of Thermal Processes on the Organoleptic and Nutraceutical Quality of Tomato Fruit

Thermal processes have a high impact on the retention of bioactive compounds in fruits and vegetables and on the bioavailability of nutraceuticals after food intake in humans [38]. This study focused on the identification of thermal processes that are able to minimize the loss of nutraceuticals and preserve the bioactivity of tomatoes.

In the present study, the increase in some organoleptic characteristics of heat-treated tomatoes was attributable to the higher time treatment, which enhanced the extraction of sugars and organic acids from plant cells, and subsequently, increased SSC and TA, respectively. The pattern is in line with the findings of Astuti et al. [39], who observed an increase in SSC after 30 and 45 min of the tomato electro-heating process when compared to 15 min of the process to obtain tomato juice. Similarly, Koltun et al. [40] noted an increase in TA percentage when analyzing tomato juice pasteurized at 121.1 °C for 42 s compared to untreated tomatoes, confirming that temperature and time are fundamental for maintaining the organoleptic quality of tomato products. Regarding color measurements, the lowest values in terms of lightness and redness found in SHS tomatoes can be related to the induction in this type of technology of the Maillard reaction. Indeed, SHS can be considered a roasting process where sugars and proteins react to obtain, through a series of reactions, browning compounds named melanoidins [41], inducing a decrease in lightness and redness of the final tomato products.

Despite the loss of color, SHS tomatoes reported interesting results in terms of the retention of bioactive compounds. Indeed, SHS increased TPC and antioxidant activity compared to fresh tomatoes, whilst lycopene and AscA contents reported similar values to fresh tomatoes. Despite its thermolability, the retention of ascorbic acid in plant foods subjected to SHS technology has already been proven in other vegetables [16,42,43]. For example, Ceccanti et al. [16] observed a similar AscA content in fresh tomatoes and tomatoes subjected to SHS treatment for 270 s and <0.15% of residual oxygen in the oven using an automatic recipe, which consisted of a variation of temperatures between 100 and 350 °C. [43] observed an increase in the AscA content of mango slices subjected to SHS treatment at 60, 70, and 80 °C for 30 min when compared to fresh mango slices. These patterns could be explained by the oxygen-free environment in the SHS chamber, which reduced AscA degradation [16]. Moreover, SHS temperature and time might have disrupted the cellular wall structure of the plant tissues, breaking the ascorbic acid cell wall bounds. This aspect can also elucidate an increase in other hydrophilic molecules, such as TPC, and the antioxidant activity of tomatoes subjected to SHS treatment (Figure 2). Indeed, the low oxygen percentage in the SHS chamber and the inactivation of the polyphenol oxidase enzyme due to high temperatures might avoid the oxidation of phenols, positively affecting the antioxidant activity. Other authors found similar patterns for the TPC and antioxidant activity results in plant food subjected to the SHS technology [17,44].

Furthermore, TPC and AscA contents have also been retained by TS treatment. The combination of the high temperatures and high duration of treatment can enhance the extraction of these compounds from plant cells, inducing patterns similar to those found for SHS treatment. In contrast, BL reduced TPC and AscA content in tomatoes (Figure 2). Other authors found similar results in plant foods [45,46,47,48]. For example, Nambi et al. [45] observed a decrease in TPC and AscA content in eggplant, green peas, green pepper, and beetroot subjected to a BL treatment at 70, 75, 80, 85, and 90 °C for 3, 6, 9, 12, and 15 min, while Mashitoa et al. [46] noted a decrease in TPC in pumpkin subjected to a 95 °C treatment for 5 min in plain water. Nambi et al. [45] showed that TPC decrease proceeded linearly with increasing temperature and the time of BL treatment. Moreover, this process released hydrophilic compounds in boiling water [49].

Regarding lycopene, the most important bioactive hydrophobic compound in tomato fruit, a retention of this molecule was observed in BL and SHS treatments when compared to fresh tomatoes, whilst TS contained reduced lycopene content. In this case, the combination of high temperatures and time induced a reverse trend to that observed for TPC and AscA content. However, the retention of this molecule in BL and SHS treatments might include different lycopene isomers characterized by different bioavailability [12]. Indeed, many examples showing the isomerization of (all-*E*)-isomers in Z-isomers using high temperatures [12,50,51] have already been reported in the literature, and the higher bioavailability of Z-isomers has also been demonstrated [12,13]. In one study, (all-*E*)-lycopene was highly purified from tomato paste and isomerized to Z-isomeric forms (5Z, 9Z, and 13Z) after thermal treatment at 120 °C for 60 min. From these isomers, the thermodynamically stable isomer (5Z)-lycopene was predominantly observed [50]. The stability of this isomer was previously demonstrated by computational results, where it showed the highest potential energy among the Z-isomers and the highest activation energy of the (all-*E*)-lycopene conversion reaction [51].

In the present study, analyzing Z-lycopene isomers, only 5Z-lycopene was observed (Figure 5), and the TS process reported a lower 5Z-lycopene content when compared with fresh and all other thermal processes. This result agrees with the total lycopene content analyzed spectrophotometrically. However, using this colorimetric assay, the lycopene content was lower compared to NMR spectroscopy due to the lower accuracy of the colorimetric method. Moreover, this result also agrees with previous studies where increasing percentages of the mono Z-isomers of lycopene, including 5Z, were observed at temperature ranges between 50 and 70 °C [14]. This fact could be related to the energy required to reach the activation energy for the isomerization process. The activation energy in kJ mol^−1^ for the isomerization reaction of (all *E*)-lycopene to 5Z-lycopene was computationally determined, and it was the highest of all the mono Z-isomers [51]. Thus, higher temperatures (100 °C) could provide the energy required to overcome the reaction energy barrier and give a higher concentration of this isomer, as shown in BL and SHS treatments in the present study. In addition to temperature, time also played an interesting role in the concentration of 5Z-lycopene. We observed that longer treatment times, as in the case of TS, resulted in lower concentrations of 5Z-lycopene, opposite to BL and SHS in which short times resulted in the highest 5Z-lycopene concentration (Figure 5). The same effect was previously observed in β-carotene from tomatoes, where after 30 min of heating at 200 °C, more than 90% of trans and cis forms were destroyed [52]. Therefore, shorter times and higher temperatures were required to produce higher quantities of 5Z-lycopene.

Oxygen is also important during isomerization processes induced by heat [53]. SHS involved the treatment in a chamber with very low oxygen concentrations. It has been reported that the degradation of lycopene showed a linear relationship with the amount of dissolved oxygen in solutions. These findings indicate that a low-oxygen environment exerts a protective effect by reducing the degradation reaction rate [54] and thus retaining lycopene as in the SHS treatment in the present study.

### 4.2. The Impact of Thermal Processes on the Preventive Effects of Tomato Fruit Extracts Against Oxidative Stress in Endothelial Cells and Vasoconstriction in Aortic Rings

Many experimental protocols have been proposed to test the antioxidant activity of nutraceuticals and dietary supplements in biological substrates. Among these, the cellular antioxidant activity (CAA) assay is one of the most described. In this series of experiments, we evaluated the preventive effects of tomato extract against a pro-oxidative stimulus, i.e., H_2_O_2_, to simulate its use prior to the development of oxidative stress and thus, to give greater “translatability” to our findings. The CAA assay is based on 2′,7′-dichlorofluorescein (DCF), which reacts not only with many intracellular reactive species, such as the hydroxyl radical and hypochlorous acid, but also with H_2_O_2_ itself. In this study, through using a fluorescent probe specific for the hydroxyl radical and not for H_2_O_2_ (i.e., DHE) to avoid possible interference, we demonstrated that fresh tomato fruit extract significantly prevents intracellular ROS production in HAECs exposed to H_2_O_2_. Conversely, tomatoes subjected to BL or TS treatments showed no beneficial effects against H_2_O_2_-induced oxidative stress. Interestingly, SHS technology maintained the antioxidant effects of fresh tomato fruit extract and significantly protected endothelial cells from oxidative damage. This result, which aligns with those obtained in the previous “cell-free” assays, confirms that SHS treatment preserves the antioxidant compounds of tomato fruit. The retention of TPC and 5Z-lycopene content in SHS-treated tomato fruit could be responsible for the observed effects. On the contrary, the reduced content of total lycopene and 5Z-lycopene in TS, as well as ascorbic acid in BL, has a negative impact on the biological activity of tomato fruit. In fact, previous studies have shown that lycopene and AscA contents improve endothelial function, minimizing H_2_O_2_-induced oxidative stress in endothelial cells [55,56,57,58,59].

Given the growing interest in the potential anti-hypertensive properties of tomatoes [6,7,8,9], the impact of thermal processes on the preventive effects of tomato fruit extract against a vasoconstrictive stimulus (i.e., NA) was evaluated in isolated rat aortic rings.

In this experimental condition, only fresh tomato extract significantly reduced NA-induced vasoconstriction, whereas heat-treated tomatoes did not reduce the vasoconstriction. This result indicates that heat may have a negative impact on the content of the bioactive compounds responsible for the preventive effects of tomato fruit against vasoconstriction, but future studies are needed to confirm this preliminary finding.

## 5. Conclusions

This study evaluated the effect of different thermal processes on the organoleptic and nutraceutical quality of tomato fruit. In addition, based on the potential effects of tomatoes in promoting vascular health, the impact of heat on the biological properties of tomato fruit was evaluated by studying the antioxidant and anti-vasoconstrictive effects of tomato fruit extracts on cultured human endothelial cells and isolated rat aortic rings, respectively.

In particular, TS, BL, and the innovative SHS technology were utilized and compared with fresh material. Despite the loss of color, SHS technology retained TPC and increased antioxidant activity compared to fresh tomatoes, whilst lycopene and AscA contents reported similar values to fresh tomatoes. In addition, SHS technology maintained the antioxidant effects of fresh tomato fruit extract and significantly protected endothelial cells from oxidative damage. This result confirms that SHS preserves bioactive compounds and maintains the biological properties of tomato fruit.

Another aspect studied in this research was the evaluation of the level of lycopene isomerization of (all-*E*)-isomers in Z-isomers. Only 5Z-lycopene was found and SHS (and BL) maintained the same level observed in fresh materials while a strong decrease in this isomer was observed following the TS treatment.

Although establishing clear trends regarding the effects of thermal processing strategies on the retention and activity of bioactive compounds in tomato fruit remains challenging, it is evident that the choice of time and temperature during thermal processes influenced these aspects and not necessarily in a negative way. However, future studies aimed at evaluating the acute effects of tomato fruit subjected to in vitro digestion and absorption, as well as the chronic effects of fresh and heat-treated tomato consumption in vivo, are essential to confirm these preliminary results and identify the most sustainable, effective, and healthy process to maintain the beneficial properties of tomato fruit on vascular health.

## Figures and Tables

**Table 1:** Tomato sauce (TS), blanching (BL), and superheated steam technique (SHS) parameters during thermal processes.

Thermal Process	Time (s)	Temperature (°C)	Relative Humidity (%)	Residual Oxygen (%)
TS	1602 ± 240	75 ± 5	91.6 ± 0.2	-
BL	10	100	100	-
SHS	420	100	93.7 ± 0.5	1.4

**Table 2:** ^1^H NMR assignments of (all-E)-lycopene isomers from fresh tomato samples.

Peak N	Chemical Shift (ppm)	Assignment	Multiplicity ^a^	J (Hz)	Reference
1	5.11	H-2 H-2′	m	-	[35,36]
2	2.11	H-3 H-3′	m	-	[35,36]
3	2.11	H-4 H-4′	m	-	[35,36]
4	5.95	H-6 H-6′	d	11	[35,36]
5	6.49	H-7 H-7′	dd	(15.1, 11.0)	[35]
6	6.25	H-8 H-8′	d	- ^b^	[35]
7	6.19	H-10 H-10′	d	11.5	[35]
8	6.63	H-11 H-11′	dd	- ^b^	[35]
9	6.36	H-12 H-12′	d	14.9	[35]
10	6.25	H-14 H-14′	m	-	[35]
11	6.61	H-15 H-15′	s	-	[35]
12	1.688	CH_3_-16 CH_3_-16′	s	-	[35,36]
13	1.615	CH_3_-17 CH_3_-17′	s	-	[35,36]
14	1.819	CH_3_-18 CH_3_-18′	s	-	[35,36]
15	1.969	CH_3_-19 CH_3_-19′	s		[35,36]
16	1.969	CH_3_-20 CH_3_-20′	s	-	[35,36]

^a^ d: doublet; dd: doublet of doublets; m: multiplet; s: singlet. ^b^: the coupling constant could not be calculated or did not match reported values because of the overlapping of signals in the mixture.

## Data Availability

The original contributions presented in the study are included in the article/Supplementary Materials, further inquiries can be directed to the corresponding author.

## Supplementary Materials

The following supporting information can be downloaded at: https://www.mdpi.com/article/10.3390/foods13223678/s1. Figure S1: Integration of signals of external standard (ethylbenzene in CDCl_3_). Figure S2: Integration region at 2.22 ppm for proton 4 of (5Z)-lycopene in all the sample replicates. Table S1: S/N ratio and Class %RSD of quantification of raw tomato fruit (Cnt) samples or subjected to sauce preparation (TS), blanching (B), and superheated steaming (SHS).
