# Supplementary material for: The Effect of Thermal Processes on the Organoleptic and Nutraceutical Quality of Tomato Fruit (Solanum lycopersicum L.)
Source: Foods. 2024 Nov 19;13(22):3678. doi: 10.3390/foods13223678 (PMC11593577; doi:10.3390/foods13223678)
Supplement: Supplementary file 1 [file foods-13-03678-s001.zip › foods-3302686-supplementary.pdf]

foods

Article

Supplementary material

The Effect of Thermal Processes on the Organoleptic and Nutraceutical Quality of Tomato Fruit (*Solanum lycopersicum* L.)

Federica Narra [1], Federico Ivan Brigante [2], Eugenia Piragine [3,4], Pavel Solovyev [2], Giada Benedetti [3], Fabrizio Araniti [5], Luana Bontempo [2], Costanza Ceccanti [1,4,*], Alma Martelli [3,4] and Lucia Guidi [1,4]

[1] Department of Agriculture, Food and Environment, University of Pisa, Via del Borghetto, 80, 56124 Pisa, Italy
[2] Traceability Unit, Research and Innovation Centre, Fondazione Edmund Mach (FEM), Via E. Mach 1, 38098 San Michele all'Adige, Italy
[3] Department of Pharmacy, University of Pisa, Via Bonanno, 6, 56126 Pisa, Italy
[4] Interdepartmental Research Center Nutrafood "Nutraceuticals and Food for Health", University of Pisa, Via del Borghetto, 80, 56124 Pisa, Italy
[5] Dipartimento di Scienze Agrarie e Ambientali—Produzione, Territorio, Agroenergia, Università degli Studi di Milano, 20133 Milan, Italy
* Correspondence: costanza.ceccanti@unipi.it

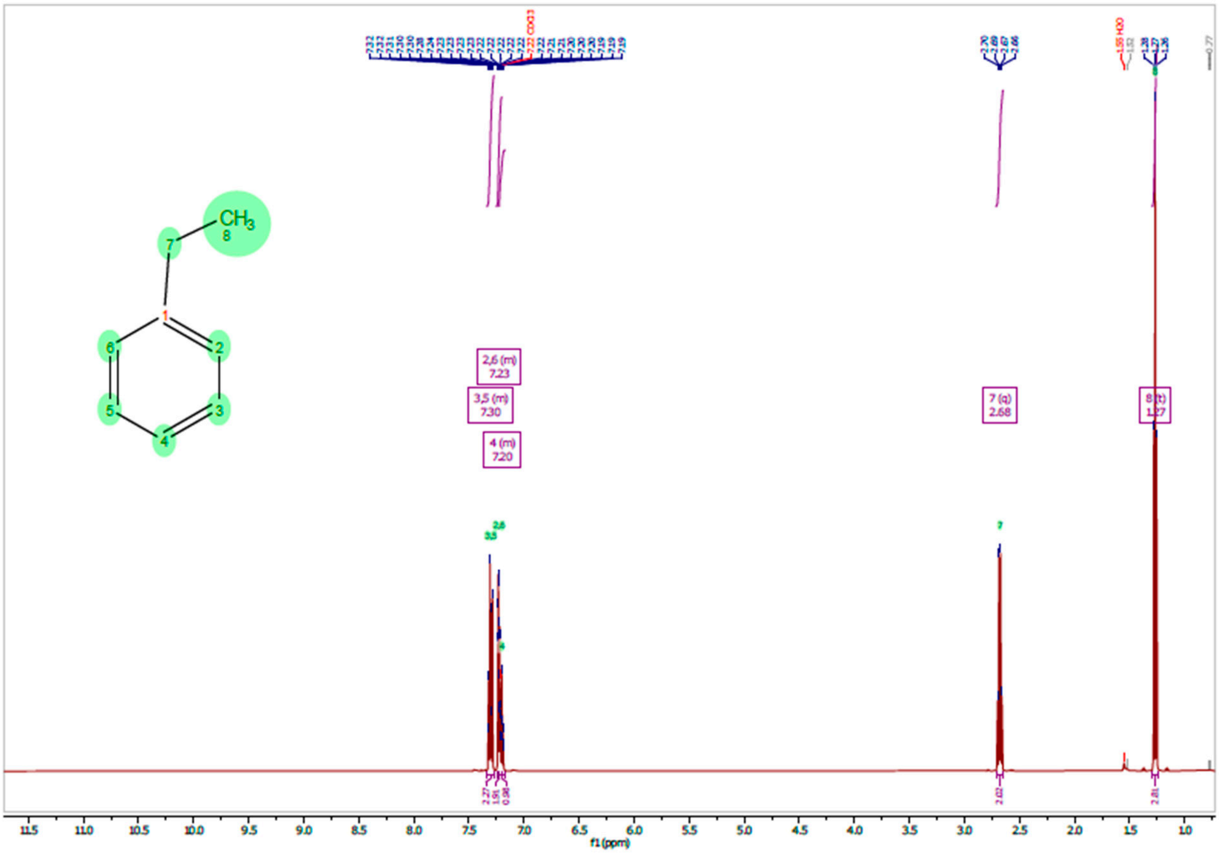

Figure S1. Integration of signals of external standard (ethylbenzene in $CDCl_3$).

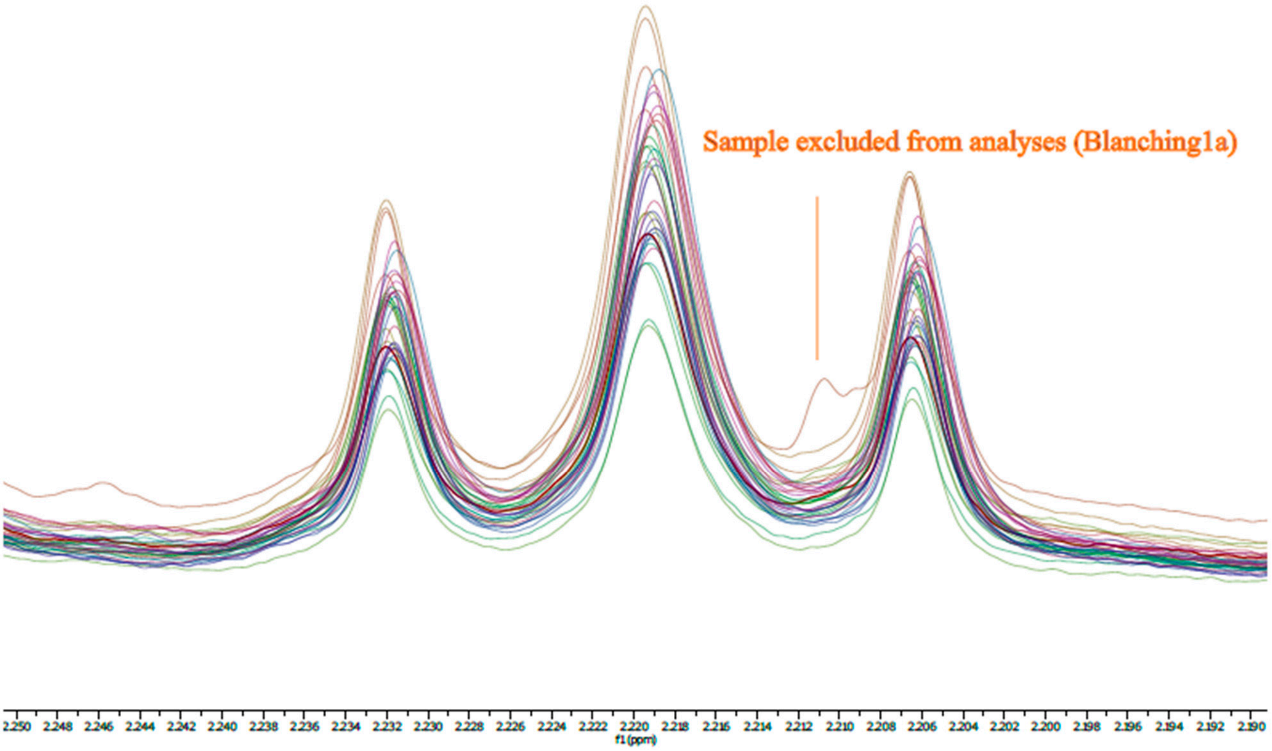

Figure S2. Integration region at 2.22 ppm for proton 4 of (5Z)-lycopene in all the sample replicates.

Table S1. S/N ratio and Class %RSD of quantification[a] of raw tomato fruit (Cnt) samples or subjected to sauce preparation (TS), blanching (B), and superheated steaming (SHS).

Sample	S/N ratio	Class-%RSD
Cnt1-a	169.3543	
**Cnt1-b**	150.9007	
Cnt1-c	229.8503	
**Cnt2-a**	239.9277	
Cnt2-b	262.249	1.0320 (n=7)
Cnt2-c	237.0557	
Cnt3-a	270.5400	
Cnt3-b	252.6730	
Cnt3-c	283.2365	
**B1-a**	235.5483	
B1-b	273.7435	
B1-c	258.8948	
B2-a	299.2254	
B2-b	306.3299	0.9392 (n=8)
B2-c	286.2372	
B3-a	298.3777	
B3-b	295.3262	
B3-c	202.2592	
TS1-a	277.3344	
TS1-b	236.6267	
TS1-c	296.9750	
TS2-a	223.0524	
TS2-b	288.7092	0.9377(n=9)
TS2-c	294.5083	
TS3-a	220.5972	
TS3-b	202.2209	
TS3-c	295.2240	
SHS1-a	225.5982	
SHS1-b	288.3946	
SHS1-c	263.0923	
SHS2-a	262.5270	
SHS2-b	256.5545	0.9425(n=9)
SHS2-c	266.9824	
SHS3-a	232.6374	
SHS3-b	208.6503	
SHS3-c	258.2322	
		Total %RSD (n=33) 0.9407

[a]Samples in bold were excluded from analysis.
